# Evolutionary biology and biodiversity research at BGRS-2018

**DOI:** 10.1186/s12862-019-1368-5

**Published:** 2019-02-26

**Authors:** Yuriy L. Orlov, Ancha V. Baranova, Nikolay A. Kolchanov, Leonid L. Moroz

**Affiliations:** 1 Institute of Cytology and Genetics SB RAS, 630090 Novosibirsk, Russia; 2 Novosibirsk State University, 630090 Novosibirsk, Russia; 3 The A.O.Kovalevsky Institute of Marine Biological Research of RAS, Moscow, Russia; 4 Research Centre for Medical Genetics, Moscow, 115478 Russia; 5 School of Systems Biology, George Mason University, Fairfax, VA USA; 6 University of Florida, Gainesville, FL USA

In this Special Issue of BMC Evolutionary Biology we present the materials from the Bioinformatics and Systems Biology summit BGRS\SB (Bioinformatics of Genome Regulation and Structure\Systems Biology) – 2018 (http://conf.bionet.nsc.ru/bgrssb2018/en/). This summit is conducted in Novosibirsk every other year, since 1998. This Special Issue accompany other BioMed Central journal issues, which highlight recent breakthroughs in the fields of genomics, bioinformatics, plant biology, and genetics, being published as BMC Genomics, BMC Bioinformatics, BMC Systems Biology, BMC Genetics, BMC Medical Genomics and BMC Plant Biology supplements [1–6]. Previous year’s highlights are described in the special issues which followed the presentations made at Belyaev Readings-2017 (http://conf.bionet.nsc.ru/belyaev100/en) [5–8].

A majority of the papers collated within current issue of BMC Evolutionary Biology were discussed at the BGRS\SB–affiliated symposium “Biodiversity: genomics and evolution” (BioGenEvo-2018). Below is a brief summary of the papers presented.

Georgy Slivko-Koltchik, Victor Kuznetsov and Yuri Panchin [9] delved into evolutionary history of gap junctions to show that the genomes of some metazoans lack both connexins and pannexins, while their 2-cell embryo’ blastomeres are electrical coupled. It seems that LRCC8 family of pannexin homologues [10] is not the final addition to happy family: gap junction treasure troves still keep some surprises to be unearthed.

Sergey Shekhovtsov et al. [11] presented a result of a challenging project – RNA-seq based assessment of the genetic lineages of five isolates of polyploid earthworm *Eisenia nordenskioldi nordenskioldi* and two outgroups from the family Lumbricidae, the congenetic *E. andrei*, and *Lumbricus rubellus*. Detailed analysis of these linages indicates complexity of earthworm evolution, with evidence of deep and ancient divergence of the lineages within *Eisenia nordenskioldi* species complex, possibly representing species which are phenotypically similar but genetically distinct.

Anastasia Kuzminkova et al. [12] presented an interactive resource which describes evolutionary analysis of epistatic interactions in protein families involved in mitochondrial function of vertebrata. In particular, they showed that rodents and primates differ in the rates of evolution for the components of various functional protein complexes and pathways.

Roman Bykov and co-authors [13] analyzed more than fifteen hundreds Paleoarctic *Drosophila* isolates for their mtDNA lineages as well as prevalence, genetic diversity and distribution patterns of their Wolbachia symbionts. Researchers concluded that the genetics structure of the population of symbionts found in subsequent fly generations is made by Wolbachia carried by the flies that survive the winter. Moreover, in study of Bykov et al., no evidences in support of so-called global Wolbachia genotype replacement hypothesis [14] were found.

Elena Kotenkova and co-authors [15] showed that the odor of mother affects the level of MRI signal in the assessor olfactory bulb neurons (AOB) to greater degree than the genetic relationships of the recipient to the donor of the odor. In related *Mus* taxa, early postnatal experiences influence subsequent choice of mates, thus, opening possibility to epigenetic origin of pre-copulatory reproductive isolation in rodent species.

Marina Rutovskaya [16] performed a comparison of the distress signals of the hybrid and parent species of the voles, created a reliable discriminator and showed that the paternal heritage has substantial influence on the sound signals observed in hybrids.

Nadezhda Bolsheva et al. [17] advanced our understanding of the evolution of the genus *Linum*, which is characterized unusual variability in size, morphology and karyotypes. The key to this diversity was found in the repeatomes of flax species. Researchers show that evolution of species comprising the genus *Linum* was accompanied by multiple waves of satellite DNA and LTR retrotransposon amplification. In fact, evolutionary tree of the *Linum* species may be built based on sole comparison of repeatomes, largely ignoring non-repetitive fraction of their genomic DNA. Note that previous publication of this research group employed high-throughput sequencing of multicopy rRNA gene families for substantial adjustments in the phylogeny of blue-flowered flax species [18].

Ksenia Strygina and Elena Khlestkina [19] analyzed structural and functional divergence of the *Mpc1* genes in wheat and barley. This study resulted in identification of regulatory R2R3-Myb genes involved in anthocyanin synthesis in Triticeae tribe species.

Taken together, this issue comprises a wide array of reports describing recent insight in evolution of various life forms, covering a spectrum from plants to invertebrates and vertebrates. At BGRS-2018, the symposium “Biodiversity: genomics and evolution” was also attended by young scientists who gathered in Novosibirsk for a School “Systems Biology and Bioinformatics” (SBB-2018) (http://conf.bionet.nsc.ru/bgrssb2018/en/school/). BioMed Central previously had published special issues by materials of SBB Schools. We invite our readers worldwide to attend our next event - Young Scientists School Systems Biology and Bioinformatics (SBB-2019) which will be held in Novosibirsk, Russia in summer of 2019 (http://conf.bionet.nsc.ru/sbb2019/en/). Evolutionary biology researches will be discussed as well at VII Congress of Vavilov Society of Geneticists and Breeders organized by of St.-Petersburg State University 18–22 June 2019 (https://events.spbu.ru/events/genetic-selection-2019). Welcome to next events!

## References

[1] Orlov YL, Baranova AV, Hofestadt R, Kolchanov NA (2016). Computational genomics at BGRS\SB-2016: introductory note. BMC Genomics.

[2] Orlov YL, Baranova AV, Salina EA (2016). Computational plant bioscience at BGRS\SB-2016: introductory note. BMC Plant Biol.

[3] Orlov YL, Baranova AV, Markel AL (2016). Computational models in genetics at BGRS\SB-2016: introductory note. BMC Genet.

[4] Baranova AV, Orlov YL. Evolutionary biology at BGRS\SB-2016. BMC Evol Biol. 2017;17(Suppl 1):21. 10.1186/s12862-016-0869-8 PMC5333185 28251876

[5] Orlov YL, Baranova AV, Hofestädt R, Kolchanov NA. Genomics at Belyaev conference - 2017. BMC Genomics. 2018;19(Suppl 3):79. 10.1186/s12864-018-4476-5 PMC5836819 29504918

[6] Orlov YL, Baranova AV, Herbeck YE. BMC Evol Biol. Evolutionary Biology at Belyaev Conference - 2017. 2017;17(Suppl 2):260. 10.1186/s12862-017-1102-0 PMC5751664 29297302

[7] Orlov YL, Baranova AV, Tatarinova TV, Kolchanov NA (2017). Genetics at Belyaev conference - 2017: introductory note. BMC Genet.

[8] Orlov YL, Moroz LL, Baranova AV (2018). Neuroscience researches at Belyaev conference-2017. BMC Neurosci.

[9] Slivko-Koltchik GA, Kuznetsov VP, Panchin YV. Are there gap junctions without connexins or pannexins? BMC Evol Biol. 2019;19(Suppl 1) (this issue, https://bmcevolbiol.biomedcentral.com/articles/supplements/volume-19-supplement-1). 10.1186/s12862-019-1369-4. 10.1186/s12862-019-1369-4 PMC6391747 30813901

[10] Abascal F, Zardoya R (2012). LRRC8 proteins share a common ancestor with pannexins, and may form hexameric channels involved in cell-cell communication. BioEssays.

[11] Shekhovtsov SV, Ershov NI, Vasiliev GV, Peltek SE. Transcriptomic analysis confirms differences among nuclear genomes of cryptic earthworm lineages living in sympatry. BMC Evol Biol. 2019;19(Suppl 1) (this issue, https://bmcevolbiol.biomedcentral.com/articles/supplements/volume-19-supplement-1). 10.1186/s12862-019-1370-y. 10.1186/s12862-019-1370-y PMC6391759 30813890

[12] Kuzminkova AA, Sokol AD, Ushakova KE, Popadin KY, Gunbin KV. mtProtEvol: the resource depositing the results of molecular evolution analysis of proteins related with vertebrate mitochondria. BMC Evol Biol. 2019;19(Suppl 1) (this issue, https://bmcevolbiol.biomedcentral.com/articles/supplements/volume-19-supplement-1). 10.1186/s12862-019-1371-x. 10.1186/s12862-019-1371-x PMC6391778 30813887

[13] Bykov RA, Yudina MA, Gruntenko NE, Zakharov IK, Voloshina MA, Melashchenko YS, Danilova MV, Mazunin IO, Ilinsky YY. Prevalence and genetic diversity of *Wolbachia* endosymbiont and mtDNA in Palearctic populations of *Drosophila melanogaster*. BMC Evol Biol. 2019;19(Suppl 1) (this issue, https://bmcevolbiol.biomedcentral.com/articles/supplements/volume-19-supplement-1). 10.1186/s12862-019-1372-9. 10.1186/s12862-019-1372-9 PMC6391860 30813886

[14] Riegler M, Sidhu M, Miller WJ, O’Neill SL (2005). Evidence for a global *Wolbachia* replacement in *Drosophila melanogaster*. Curr Biol.

[15] Kotenkova E, Romachenko A, Ambaryan A, Maltsev A. Effect of early experience on neuronal and behavioral responses to con- and heterospecific odors in closely related Mus taxa: epigenetic contribution in formation of precopulatory isolation. BMC Evol Biol. 2019;19(Suppl 1) (this issue, https://bmcevolbiol.biomedcentral.com/articles/supplements/volume-19-supplement-1). 10.1186/s12862-019-1373-8. 10.1186/s12862-019-1373-8 PMC6391773 30813903

[16] Rutovskaya M. Inheritance of the acoustic signal parameters in interspecies hybrids of the Bank (*Myodes glareolus*) and the Tien Shan (*M. centralis*) voles**.** BMC Evol Biol. 2019;19(Suppl 1) (this issue, https://bmcevolbiol.biomedcentral.com/articles/supplements/volume-19-supplement-1). 10.1186/s12862-019-1374-7. 10.1186/s12862-019-1374-7 PMC6391760 30813911

[17] Bolsheva NL, Melnikova NV, Kirov IV, Dmitriev AA, Krasnov GS, Amosova AV, Samatadze TE, Yurkevich OY, Zoshchuk SA, Kudryavtseva AV, Muravenko OV. Characterization of repeated DNA sequences in genomes of blue-flowered flax BMC Evol Biol. 2019;19(Suppl 1) (this issue, https://bmcevolbiol.biomedcentral.com/articles/supplements/volume-19-supplement-1). 10.1186/s12862-019-1375-6. 10.1186/s12862-019-1375-6 PMC6391757 30813893

[18] Bolsheva NL, Melnikova NV, Kirov IV, Speranskaya AS, Krinitsina AA, Dmitriev AA, Belenikin MS, Krasnov GS, Lakunina VA, Snezhkina AV, Rozhmina TA, Samatadze TE, Yurkevich OY, Zoshchuk SA, Amosova АV, Kudryavtseva AV, Muravenko OV (2017). Evolution of blue-flowered species of genus Linum based on high-throughput sequencing of ribosomal RNA genes. BMC Evol Biol.

[19] Strygina K, Khlestkina E. Structural and functional divergence of the *Mpc1* genes in wheat and barley. BMC Evol Biol. 2019;19(Suppl 1) (this issue, https://bmcevolbiol.biomedcentral.com/articles/supplements/volume-19-supplement-1). 10.1186/s12862-019-1378-3. 10.1186/s12862-019-1378-3 PMC6391766 30813913

